# Supplementary material for: Causal interplay between lactose intolerance and gut microbiota: a combined bidirectional Mendelian randomization and in vivo validation study
Source: Front Nutr. 2026 Jun 1;13:1803337. doi: 10.3389/fnut.2026.1803337 (PMC13265576; doi:10.3389/fnut.2026.1803337)
Supplement: Supplementary file 1 [file Data_Sheet_1.zip › supplementary materials/Forward/leave one out cross validation/ebi-a-GCST90027644.finngen_R12_E4_LACTONAS.pdf]

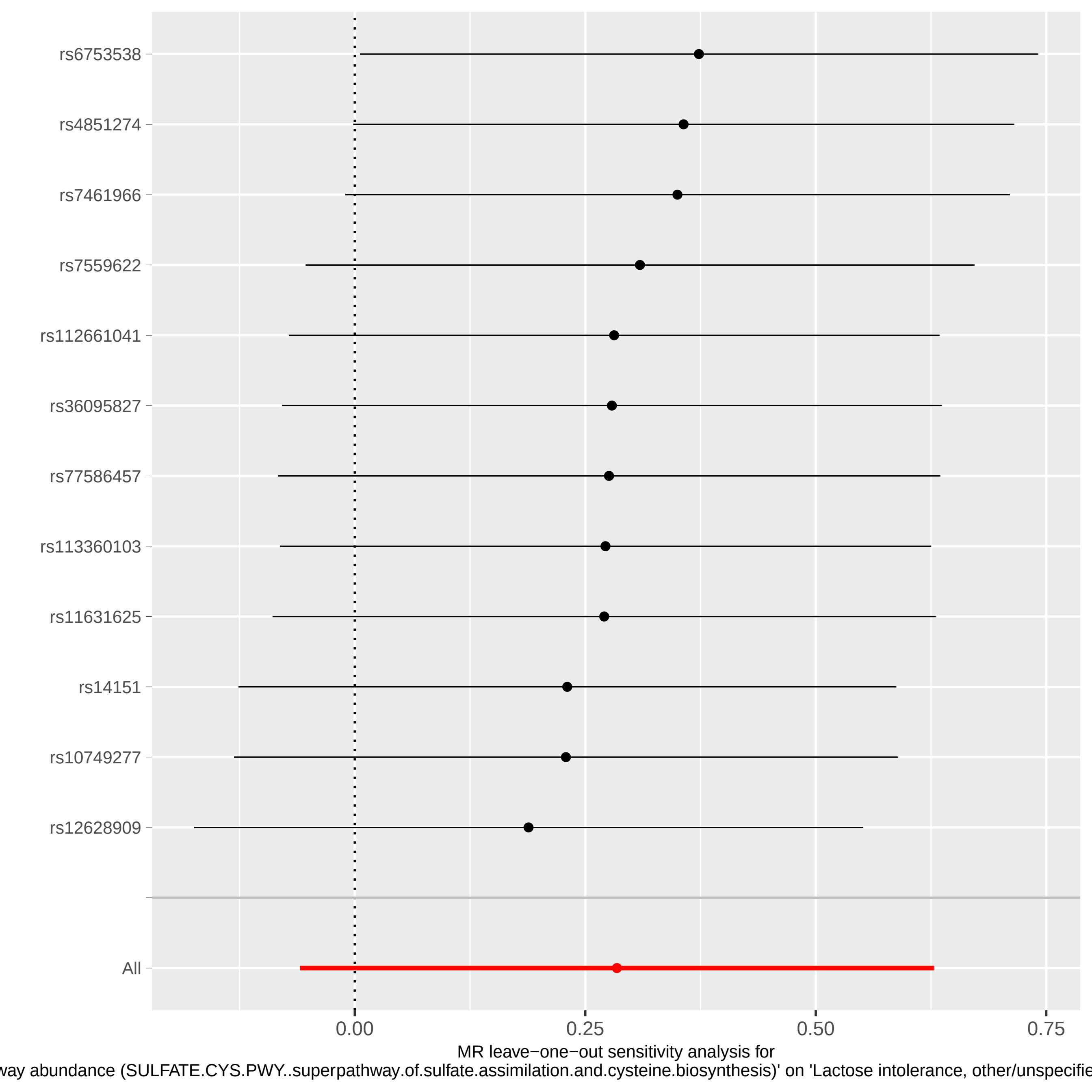
rs6753538
rs4851274
rs7461966
rs7559622
rs112661041
rs36095827
rs77586457
rs113360103
rs11631625
rs14151
rs10749277
rs12628909
All
0.00
0.25
0.50
0.75
MR leave−one−out sensitivity analysis for
way abundance (SULFATE.CYS.PWY..superpathway.of.sulfate.assimilation.and.cysteine.biosynthesis)' on 'Lactose intolerance, other/unspecifie
